# Supplementary material for: Altered Cellular Metabolism Is a Consequence of Loss of the Ataxia-Linked Protein Sacsin
Source: Int J Mol Sci. 2024 Dec 10;25(24):13242. doi: 10.3390/ijms252413242 (PMC11675909; doi:10.3390/ijms252413242)
Supplement: Supplementary file 1 [file ijms-25-13242-s001.zip › ijms-3325270-supplementary.pdf]

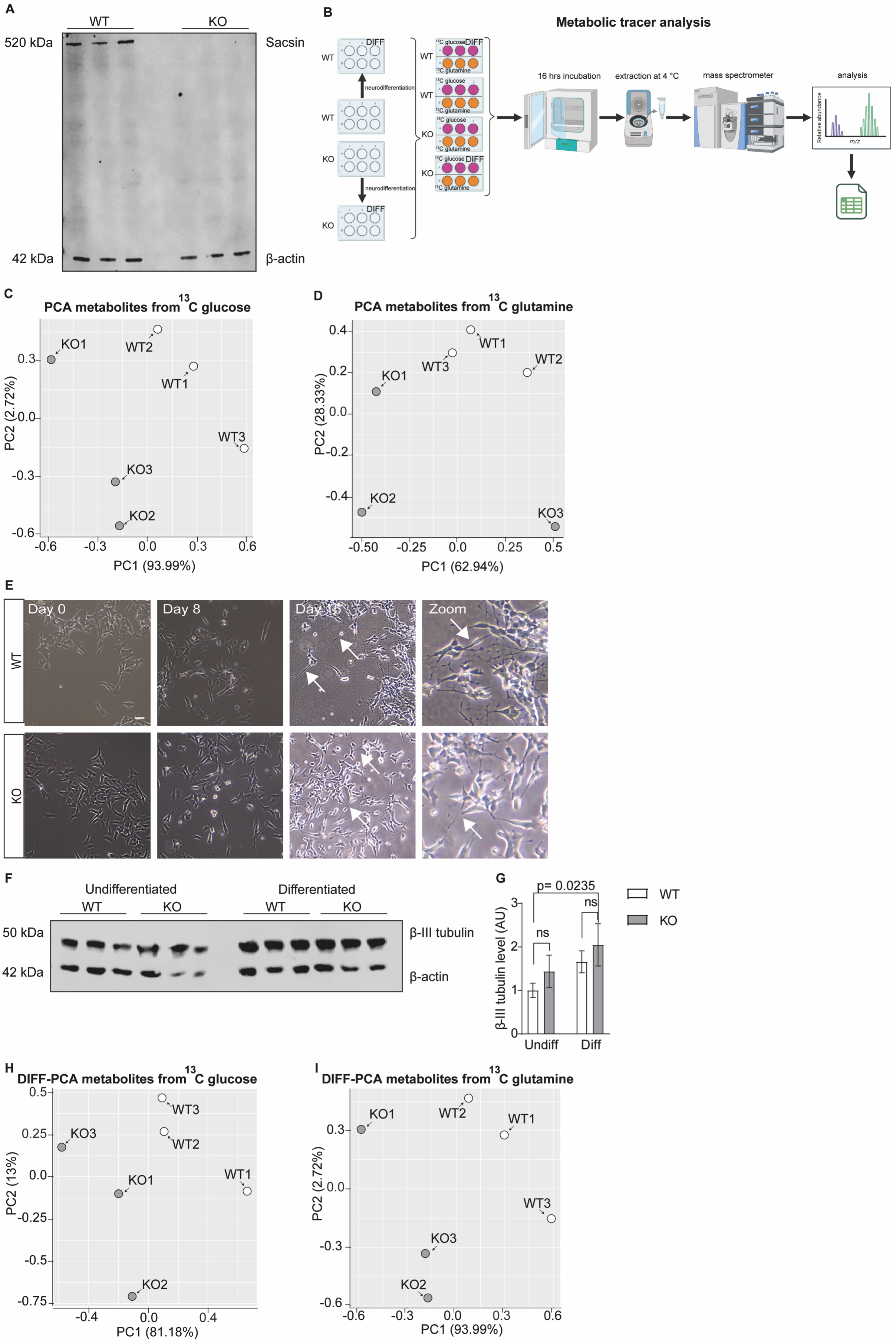

A
WT
KO
520 kDa
Sacsin
42 kDa
β-actin
B
Metabolic tracer analysis
DIFF
neurodifferentiation
16 hrs incubation
extraction at 4 °C
mass spectrometer
analysis
Relative abundance
m/z
C
PCA metabolites from 13C glucose
PC2 (2.72%)
PC1 (93.99%)
D
PCA metabolites from 13C glutamine
PC2 (28.33%)
PC1 (62.94%)
E
Day 0
Day 8
Day 15
Zoom
F
Undifferentiated
Differentiated
50 kDa
β-III tubulin
G
p= 0.0235
ns
β-III tubulin level (AU)
Undiff
Diff
H
DIFF-PCA metabolites from 13C glucose
PC2 (13%)
PC1 (81.18%)
I
DIFF-PCA metabolites from 13C glutamine
PC2 (2.72%)
PC1 (93.99%)

Supplementary Figure S1. PCA analysis of metabolomic data from wild-type control and sacsin knockout cells. (A) Immunoblot of sacsin in SH-SY5Y wild-type control (WT) and SH-SY5Y knockout (KO) lysates. (B) Flowchart illustrating the design of the metabolic tracer analysis experiment.(C-D) Principal component analysis (PCA) from three sacsin knockout clones and control cells of (C) traced glucose metabolites, (D) traced glutamine metabolites. (E) Representative images of wild-type control and sacsin knockout SH-SY5Y cells through neuronal differentiation. Arrows indicate neurite. Scale bar 5 μm. (F) Immunoblot of β-III tubulin and actin (loading control) in both undifferentiated and differentiated wild-type control and sacsin knockout cells. (G) Densitometry of the immunoblot E. Unpaired t-test. (H,I) Principal component analysis (PCA) from three differentiated sacsin knockout clones and control cells of (H) traced glucose metabolites and (I) traced glutamine metabolites. WT= wild-type control, KO= sacsin knockout, Undiff = undifferentiated SH-SY5Y, diff = differentiated SH-SY5Y.

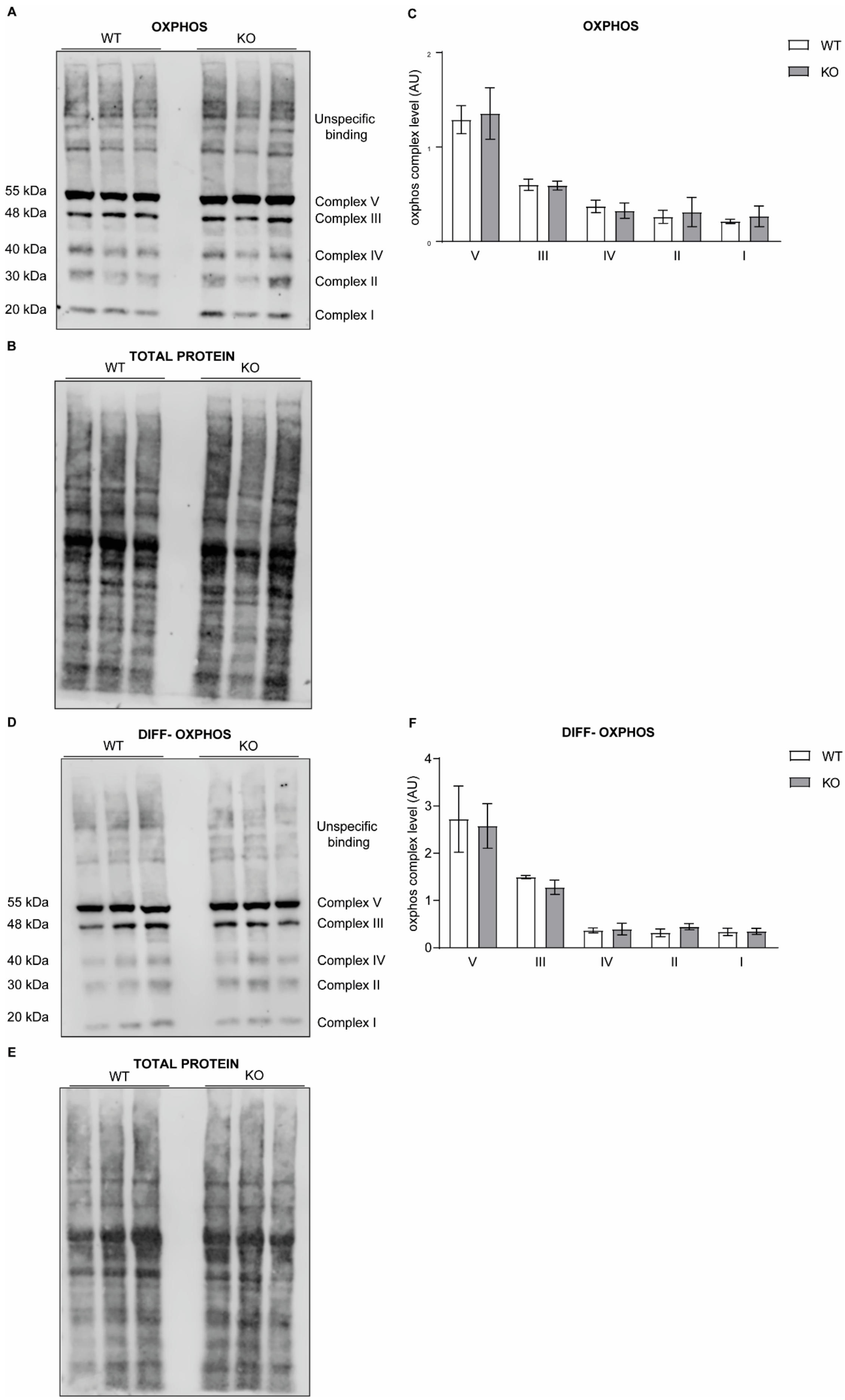
A
OXPHOS
WT
KO
Unspecific binding
55 kDa
48 kDa
40 kDa
30 kDa
20 kDa
Complex V
Complex III
Complex IV
Complex II
Complex I
B
TOTAL PROTEIN
WT
KO
C
OXPHOS
WT
KO
oxphos complex level (AU)
2
1
0
V
III
IV
II
I
D
DIFF- OXPHOS
WT
KO
Unspecific binding
55 kDa
48 kDa
40 kDa
30 kDa
20 kDa
Complex V
Complex III
Complex IV
Complex II
Complex I
E
TOTAL PROTEIN
WT
KO
F
DIFF- OXPHOS
WT
KO
oxphos complex level (AU)
4
3
2
1
0
V
III
IV
II
I

Supplementary Figure S2. Electron transport chain cycle complexes were not altered in sacsin knockout cells. (A) Immunoblot of OXPHOS in SH-SY5Y wild-type control (WT) and SH-SY5Y knockout (KO) lysates. (B) Total protein lysates detected with fast green staining. (C) Densitometry of the OXPHOS immunoblot. (D) Immunoblot of OXPHOS in differentiated SH-SY5Y wild-type control (WT) and SH-SY5Y knockout (KO) lysates. (E) Differentiated total protein lysates detected with fast green staining. (F) Densitometry of the differentiated OXPHOS immunoblot.

Unpaired t-test. n=3. S.D. error bars. WT= wild-type control, KO= sacsin knockout, Undiff = undifferentiated SH-SY5Y, diff = differentiated SH-SY5Y.
